# Tube ileostomy technique successfully preserved the ileocecal region in a case of fetal intestinal volvulus without malrotation: A case report

**DOI:** 10.1016/j.ijscr.2025.112121

**Published:** 2025-10-27

**Authors:** Kohei Kawaguchi, Seiichiro Inoue, Yuki Muta, Yuta Takeuchi, Akio Odaka

**Affiliations:** a Department of Hepato-Biliary-Pancreatic Surgery and Pediatric Surgery, Saitama Medical Center, Saitama Medical University, 1981 Kamoda, Kawagoe, Saitama, 350-8550, Japan

**Keywords:** Ileocecal preservation, intestinal volvulus without malrotation, tube ileostomy, Case report

## Abstract

**Introduction and importance:**

Preserving the ileocecal valve in terminal ileal necrosis is crucial to prevent bile acid malabsorption and growth impairment. Fetal intestinal volvulus without malrotation (IVWM) is rare. This report highlights successful valve preservation via tube ileostomy in a neonate with IVWM, offering practical insight into neonatal bowel management.

**Presentation of case:**

A male neonate was delivered by emergency cesarean section at 37 weeks due to antenatal bowel dilation. A whirlpool sign on ultrasound prompted laparotomy, revealing a 1080-degree volvulus 2 cm distal to the ileocecal valve without malrotation. Thirty-two centimeters of necrotic ileum were resected. Due to inflammation, anastomosis was not feasible; an end ileostomy and distal tube ileostomy were performed, preserving 55 cm of proximal bowel and 2 cm distal to the valve. Weight gain improved following parenteral nutrition and stool recycling. Contrast study on day 53 confirmed distal patency, and reanastomosis was completed on day 94. The patient was discharged 47 days later.

**Clinical discussion:**

When immediate anastomosis is not feasible, distal closure is often chosen. This case shows that tube ileostomy with stool recycling can preserve function even with minimal distal ileum, supporting growth and adaptation.

**Conclusion:**

Tube ileostomy enabled valve preservation and a favorable outcome.

## Highlights

• Tube ileostomy is valuable when anastomosis must be delayed.
• Stool recycling supports nutritional recovery.
• Prenatal ultrasound aids early detection and intervention in fetal volvulus.

## Graphical abstract

**Figure:**
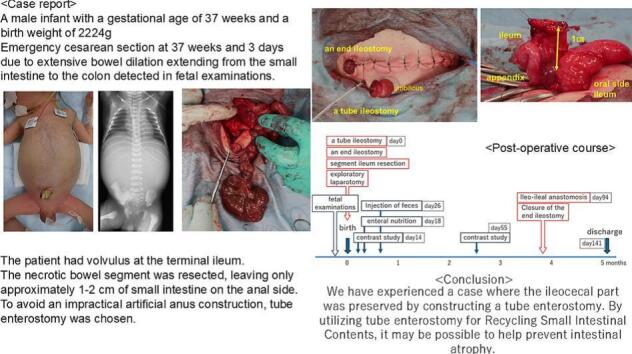

## 1 Introduction

Fetal intestinal volvulus without malrotation (IVWM) is an extremely rare condition, with fewer than 100 cases reported worldwide.

Zhou et al. described a series of seven prenatally diagnosed cases [1]. In addition, a nationwide survey in Germany estimated the incidence of IVWM in very low birthweight infants at approximately 1–1.5 per 1000 live births [2].

In most cases, intestinal volvulus in infants and children is midgut volvulus associated with malrotation [3], and IVWM in infants is relatively rarer [4]. Prenatal or presurgical diagnosis can be very difficult. Moreover, fetal IVWM requires emergency neonatal surgery, which often includes resection of the necrotic intestine. Various regions of the small intestine may be involved in segmental torsion. When the terminal ileum is involved, ileocecal resection may be necessary. However, this procedure may result in nutrient malabsorption, potentially leading to growth impairment.

We encountered a case of fetal IVWM involving the terminal ileum. In this case, we were able to preserve the ileocecal region using the tube ileostomy technique. The patient's clinical course was favorable after beginning stool recycling, and he was discharged from our center after stoma closure. Here, we describe the clinical course and intraoperative findings of this IVWM case. This case report has been reported in line with the SCARE checklist [include citation].

## 2 Presentation of case

**Fig. 1:**
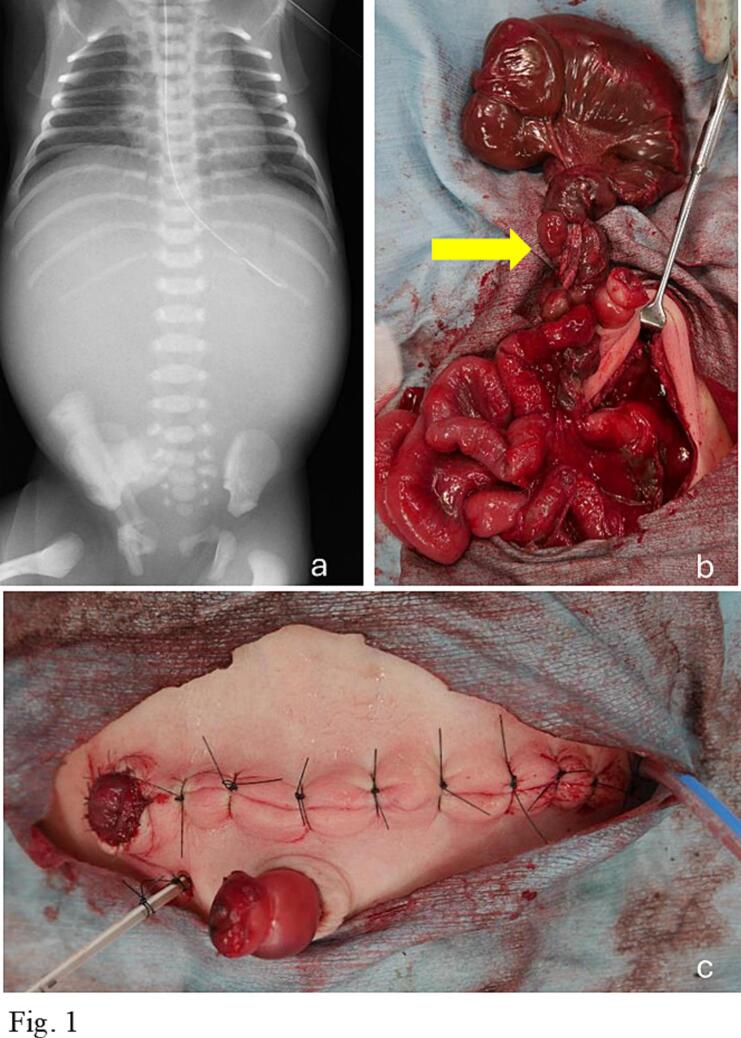
a: Abdominal X-ray without intestinal gas or free air. b: Intraoperative findings: A small bowel volvulus was found 2 cm distal to the ileocecal valve with a 1080-degree rotation. The yellow arrow (↓) shows the pedicle of the intestinal volvulus. c: Postoperative findings: A proximal end ileostomy and a distal tube ileostomy were created. A 6 Fr feeding tube was placed in the ascending colon. (For interpretation of the references to colour in this figure legend, the reader is referred to the web version of this article.)

**Fig. 2:**
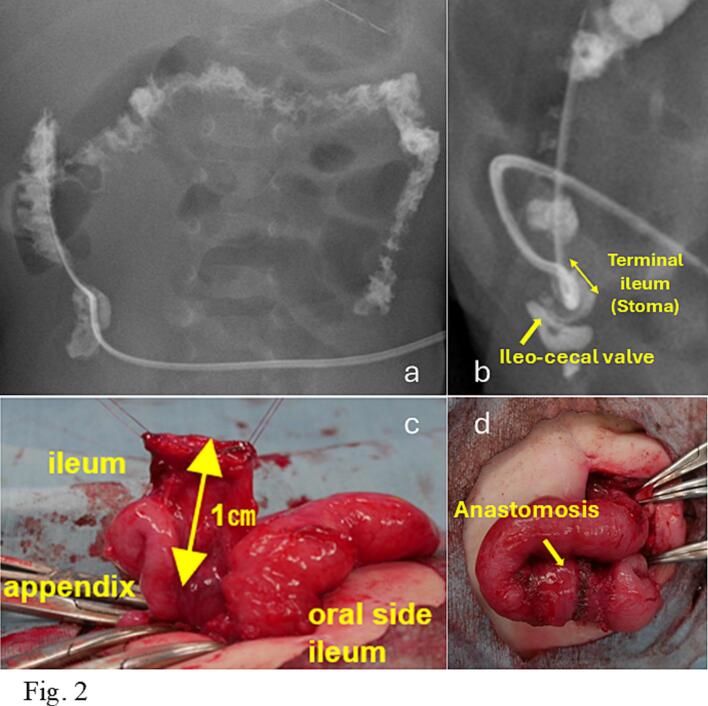
a: A stoma contrast study performed on postnatal day 53. b: The stoma contrast study demonstrated a normal cecal distance from the skin (approximately 2 cm), with no evidence of colonic narrowing or decreased bowel diameter. Contrast agent injected through the tube flowed from the cecum into the distal ileum and was excreted through the stoma. c: Closure of the ileostomy. At closure, approximately 1 cm (yellow arrow) of terminal ileum was able to be preserved, and an ileoileal anastomosis was performed. d: The lumen diameter at the ileoileal anastomotic site was comparable to that of the adjacent intestine. (For interpretation of the references to colour in this figure legend, the reader is referred to the web version of this article.)

A boy was diagnosed with extensive intestinal dilatation and fetal ascites by fetal ultrasonography at 37 weeks and 2 days of gestation and delivered by emergency cesarean section the next day. His birth weight was 2224 g, and his Apgar score was 5/9. The abdomen was distended and tense with no discoloration. Abdominal x-ray showed no intestinal gas or free air (Fig. 1a). A small intestinal whirlpool sign was observed on post-birth ultrasonography, prompting an emergency laparotomy. The procedure was performed under general anesthesia with the patient in the supine position. MPEM was administered as the perioperative antibiotic. A 6 cm abdominal transverse incision was made one fingerbreadth above the umbilicus. Upon exploration of the entire small intestine, a small intestinal volvulus was identified 2 cm distal to the ileocecal valve, with a 1080-degree counterclockwise rotation. No Ladd's bands were present, and the positions of the small and large intestines were normal. Based on these findings, intestinal malrotation was excluded, and a diagnosis of isolated volvulus without malrotation (IVWM) was made (Fig. 1b). A 32 cm segment of necrotic ileum was resected, and approximately 2 cm of terminal ileum was able to be preserved. The ileocecal region and surrounding tissues were severely adherent and could not be elevated from the abdominal wall. Although the ileocecal valve was identifiable, the appendix was presumed to be located dorsally and could not be clearly visualized. While a double-barrel stoma was considered, an end ileostomy was created proximally, and a tube ileostomy was established at the terminal ileum. A 6 Fr feeding tube was inserted into the ascending colon and secured with purse-string sutures (Fig. 1c). Due to the distance between the ileocecal region and the abdominal incision, fixation by purse-string suture alone posed a high risk of accidental dislodgement. Therefore, the tube was inserted and retained within the ascending colon. Postoperatively, enteral feeding gradually increased, but weight gain was poor. Total parenteral nutrition with ELENTAL Combination Powder® (EA Pharma Co., Ltd., Japan) was initiated to improve weight gain. The stool recycling protocol was initiated on postoperative day 15 with continuous infusion of SOLITA-T GRANULES No.3® (AY PHARMACEUTICALS CO., LTD., Tokyo, Japan) solution at 1 ml/h, which was subsequently increased to 2 ml/h. From postoperative day 24, the infusion fluid was switched to Small Intestinal Contents, which were reinfused once daily over an 8-hour period. The protocol was intermittently suspended and resumed according to the patient's general condition. A stoma contrast study performed on postnatal day 53 showed an approximately 2 cm distance from the skin to the cecum, with no colonic narrowing and preserved bowel diameter. Contrast agent injected from the tube tip in the cecum flowed into the distal ileum and was excreted through the stoma (Fig. 2a, b).

Closure of the ileostomy was planned for postnatal day 94. At closure, approximately 1 cm of terminal ileum was able to be preserved, and an ileoileal anastomosis was performed (Fig. 2c, d). Postoperatively, the infant transitioned from ELENTAL Combination Powder® to breast milk and showed improvement. Weight gain was good, and the infant was discharged on postoperative day 47. No adverse events per Clavien-Dindo classification. At the 9-month postoperative follow-up, the patient demonstrated satisfactory weight gain and maintained a stool frequency of at least once daily. Vitamin B12 levels were not assessed, as this vitamin is generally stored in the liver and deficiency was not anticipated within this period. The patient's caregivers expressed deep gratitude, and outpatient follow-up is still ongoing.

## 3 Discussion

IVWM can occur in any part of the intestine, as elongation or formation of a mesenteric pedicle due to mesenteric narrowing is considered a possible cause [5]. In this case, we opted for a tube enterostomy rather than forcibly creating a distal stoma. Alternative procedures, such as Bishop–Koop ileostomy or simple distal closure, are sometimes considered in neonates with short bowel. Bishop–Koop ileostomy enables early assessment of distal bowel function, whereas distal closure is technically simpler but may increase the risk of distal obstruction. Tube ileostomy, in contrast, provides decompression and protection of the distal bowel but carries potential risks, including tube dislodgement and infection. In the present case, tube ileostomy was successful because 55 cm of proximal small intestine was preserved and the luminal diameter of the remaining bowel was adequate (30–40 mm), which allowed effective decompression and preservation of continuity. This approach was intended to promote bowel rest and growth, which we believe facilitated the subsequent bowel anastomosis. IVWM is typically diagnosed in the fetal or immediate neonatal period, and urgent delivery and postnatal surgery are necessary when intestinal volvulus is suspected on a fetal ultrasound [6]. In our case, the diagnosis was made at 37 weeks of gestation, relatively close to term, and the patient underwent an emergency cesarean section followed immediately by surgery, which we believe contributed to the favorable outcome.

Prenatal ultrasound plays a pivotal role in the suspicion of fetal intestinal volvulus; however, its diagnostic sensitivity is limited. In a recent series, whirlpool or vascular spiral signs were detected in 20 of 27 examinations (approximately 74 %), underscoring that these findings are not consistently present [1]. Therefore, careful interpretation in combination with clinical context is essential for accurate diagnostic reasoning.

As Venous Thromboembolism (VTE) prophylaxis is not applicable in neonates, we focused on infection prevention during total parenteral nutrition (TPN) management. In the neonatal intensive care unit (NICU), we closely monitored the patient with regular blood tests and vital sign assessments to detect any potential line-related infections.

The ileocecal valve is considered a key functional component of the terminal ileum, as it allows intestinal content to remain in the terminal ileum briefly, promoting reabsorption of bile salts, chyme, and nutrients [7]. It also prevents reflux of contents from the colon into the small intestine, reducing the risk of bacterial overgrowth and intestinal infection [8]. Although studies have reported that ileocecal resection in pediatric patients does not affect long-term growth or prognosis and is not associated with increased morbidity and mortality [9], many authors advocate for preserving the ileocecal junction, especially the ileocecal valve, whenever possible [10]. For example, Yan et al. [10] reported that ileocecal resection in infants with intestinal duplication had no effects on long-term growth or defecation patterns. However, in patients with short bowel syndrome, the presence or absence of the ileocecal valve affects the minimum length of small intestine required for survival [ 11]. Loss of this valve may also predispose patients to colonic content reflux, small intestinal inflammation, or recurrence of Crohn's disease after surgical treatment. Moreover, even in the absence of short bowel syndrome or Crohn's disease, ileocecal resection can lead to early postsurgical complications such as diarrhea, acid-base imbalance, fluid and electrolyte imbalances, impaired reflux control, accelerated intestinal transit time, and reduced intestinal absorption [7,12]. These potential complications underscore the importance of ileocecal preservation, especially in neonates and infants requiring surgery for terminal ileal disease. In this case, preservation of the cecum was crucial in preventing these complications and achieving a favorable clinical outcome. In the long term, preservation of the ileocecal valve is particularly important in neonates with short bowel syndrome, as its presence has been associated with a 20–30 % reduction in TPN dependence [13]. This functional advantage is attributed to delayed intestinal transit, improved absorption of bile salts and nutrients, and prevention of bacterial overgrowth. Beyond IVWM, tube ileostomy may also be applicable in other neonatal conditions where preservation of distal bowel continuity is desirable, such as after extensive small bowel resection, intestinal atresia, traumatic bowel injury, or allied disorders of Hirschsprung's disease.

This report has several limitations. As a single case from a Japanese cohort, its generalizability to more diverse populations, particularly those with different levels of access to prenatal care, may be limited. Nevertheless, the case highlights important strengths, including its multidisciplinary relevance across neonatology and radiology, which may provide useful insights for clinicians managing similar conditions.

## 4 Conclusion

In this case of fetal IVWM affecting the terminal ileum, effective use of tube ileostomy (with the tip inserted into the ascending colon) and stool recycling helped preserve the ileocecal region. Traditionally, terminal ileal torsion is managed by simply closing the distal end and returning the bowel to the abdominal cavity. However, because only a very short segment of the terminal ileum could be preserved, we opted for a tube enterostomy with the tip placed in the cecum to facilitate stool recycling. This approach appears to have promoted growth of the terminal ileum and contributed to ileocecal preservation.

## Abbreviations

IVWM: intestinal volvulus without malrotation
VTE venous thromboembolism
TPN: total parenteral nutrition

## Author contribution

Kohei Kawaguchi contributed to the study concept, design, and manuscript writing.

Seiichiro Inoue provided academic consultation and critically revised the manuscript for language and content.

Yuki Muta and Yuta Takeuchi were responsible for clinical data collection and organization.

Akio Odaka supervised the overall project and provided senior oversight.

All authors have read and approved the final version of the manuscript.

## Patient consent

Ethics approval and consent were waived because this case report is a review of literature with a retrospective case report on one patient. The patient gave consent to participate for publication. Written informed consent was obtained from the patient's parents/legal guardian for publication and any accompanying images. A copy of the written consent is available for review by the Editor-in-Chief of this journal on request. This report contains no personal information that could identify the patient.

## Ethical approval

This case report is exempt from ethical approval according to the institutional policy of Yokohama General Hospital, as it involves a single anonymised patient with informed consent.

## Guarantor

Kohei Kawaguchi.

## Research registration number

The intervention presented in this case report has previously been described in human subjects and does not constitute a “First in Man” study. As such, registration was not applicable for this report.

## Funding

This research did not receive any specific grant from funding agencies in the public, commercial, or not-for-profit sectors.

## Conflict of interest statement

The authors declare that they have no competing financial interests or personal relationships that may have influenced the work reported in this study.
